# Bio-Guided Fractionation of *Retama raetam* (Forssk.) Webb & Berthel Polar Extracts

**DOI:** 10.3390/molecules26195800

**Published:** 2021-09-24

**Authors:** Mariem Saada, Hanen Wasli, Inès Jallali, Rim Kboubi, Karl Girard-Lalancette, Vakhtang Mshvildadze, Riadh Ksouri, Jean Legault, Susana M. Cardoso

**Affiliations:** 1 Centre de Biotechnologie de Borj-Cédria (LR15CBBC06), Laboratoire des Plantes Aromatiques et Médicinales, BP 901, Hammam Lif 2050, Tunisia; saadamariem@gmail.com (M.S.); hanenwasli@gmail.com (H.W.); phytochimie2009@yahoo.com (I.J.); ksouririadh@gmail.com (R.K.); 2 Faculté des Sciences de Tunis, Université de Tunis El Manar, Tunis 2092, Tunisia; 3 Laboratoire LASEVE, Université du Québec à Chicoutimi, 555 Boulevard de l’Université, Chicoutimi, QC G7H 2B1, Canada; rimkboubi@yahoo.fr (R.K.); karl_girard-lalancette@uqac.ca (K.G.-L.); vakhtang_mshvildadze@uqac.ca (V.M.); jean_legault@uqac.ca (J.L.); 4 LAQV-REQUIMTE & Department of Chemistry, University of Aveiro, 3810-193 Aveiro, Portugal

**Keywords:** *Retama raetam*, open column chromatography, phenolic compounds, LC-DAD-ESI/MS^n^, antioxidant, cytotoxicity

## Abstract

The fractionation of the methanolic extract (MeOH-E) of *Retama raetam* (Forssk.) Webb & Berthel and further analysis by thin layer chromatography resulted in four fractions (F1, F2, F3 and F4) that, in parallel with the MeOH-E, were screened for antioxidant, cytotoxic, antidiabetic and antibacterial properties. In addition, chemical characterization of their bioactive molecules was performed using LC-DAD-ESI/MS^n^. The results indicated that F3 was the most promising regarding antioxidant and cytotoxicity abilities, possibly due to its richness in flavonoids class, particularly isoflavones. In turn, F1 was characterized by the presence of the most polar compounds from MeOH-E (organic acids and piscidic acid) and showed promising abilities to inhibit α-amylase, while F4, which contained prenylated flavonoids and furanoflavonoids, was the most active against the tested bacteria. The gathered results emphasize the distinct biological potentials of purified fractions of *Retama raetam*.

## 1. Introduction

Aromatic plants, also known as herbs and spices, have been used since antiquity as folk medicine and as preservatives in foods. They contain various biologically active compounds, including phenolic compounds, which are closely associated with a range of health benefits such as antimicrobial, antioxidant, antiparasitic and anti-inflammatory activities [1]. The demand for these plants and their derivatives has increased since consumers seek for more natural products, which in general are considered eco-friendly and safe products. Therefore, aromatic plants and their extracts have the potential to become new generation substances for human and animal nutrition and health [1].

The Retama genus (Fabaceae, subfamily Faboideae, tribu Genisteae) comprises four species that are mainly distributed in the Mediterranean Basin. These plants have been used traditionally for the treatment of different diseases in many parts of the Mediterranean Basin, of which *R. raetam* is the most applied [2]. *R. raetam* (Forsk.) Webb., also known as white broom or white weeping broom, is a desert shrub native to several countries of North Africa, including Morocco, Algeria, Tunisia, Libya, and Egypt, temperate Asia and in certain Middle Eastern countries (e.g., Palestine, Lebanon, Jordan and Israel) [3]. The distinct parts of this plant, namely cladodes (photosynthetic stems), flowers, seeds and roots, are included in the pharmacopoeia and are commonly employed as a powder, in an infusion or decoction for external use such as a cataplasm or poultice, as a bath or for oral use [2]. The medicinal application of *R. raetam* has, in part, been supported by scientific data. In this regard, it is of note the fact that distinct authors reported in vivo health benefits of *R. raetam* polar extracts using animal models. In particular, Al-Tubuly and colleagues [4] evaluated the effect of a methanol (MeOH) extract from the aerial parts of *R. raetam* on the central nervous system (CNS), highlighting variable effects on anxiety levels and behaviors of mice at different doses. Moreover, the hepatoprotective effects of a hydromethanolic extract from *R. raetem* seeds were evidenced by Omara et al. [5], who described the ability to reduce liver changes induced in rats by CCl_4_. Furthermore, Maghrani and coworkers [6] demonstrated the significant diuretic potential of aqueous extract from the aerial parts of the plant in normal rats. The same group of authors also noted the ability of *R. raetem* to control glucose and/or lipid metabolism in normal and/or in STZ diabetic rats using polar extracts. The hypoglycemic ability was described either for a MeOH extract from fruits, for aqueous extracts obtained from leaves and from the whole plant [7], while the potential to decrease plasma cholesterol and triglycerides levels was demonstrated for the latter extract [8]. In addition to the benefits described in animal models, distinct authors claimed other promising effects of *R. raetem* polar extracts, of which antimicrobial and antioxidants properties are commonly cited [2].

Regardless, most authors claim that the pharmacological activity of this plant is associated to its richness in phytochemicals, particularly flavonoids and alkaloids [2], yet there is a huge lack of information in most studies concerning phytochemical constituents of the tested extracts. In fact, the above cited in vivo studies contain none or only minimal chemical information of the tested extracts [2]. Moreover, despite the considerable studies focusing on antimicrobial/antioxidant abilities of polar extracts from distinct parts of *R. raetem*, most of them only associated such abilities with their richness in groups of compounds, namely phenolics [9,10,11,12,13]. To our knowledge, it was only recently that the lack of correlation between bioactivity and phenolic profile of *R. raetem* extracts started to be overcome. This is a key step not only for settling understanding possible health applications of each extract, as well as to help on the search for best extract conditions when having in mind future standardization/commercialization [2]. As known, the potential bioactivity of each extract is much dependent on its phytochemical composition and, in turn, this varies greatly according to distinct factors, including the growth environmental conditions of the plant, the parts of the plant material used, the plant maturation stage, harvesting/drying methods and the extracting conditions applied (e.g., method, solvent, temperature and time) [14].

As mentioned, information on the chemical composition of *R. raetem* polar extracts and/or of purified fractions is scarce. The use of HPLC analysis previously allowed some members of our group to identify syringic acid and coumarin as major components on a purified fraction (obtained from a hydroacetonic crude extract) from *R. raetem* cladodes, which exhibited potential antioxidant and antimicrobial abilities [15]. Few authors have so far applied the UHPLC-DAD-ESI-MS^n^ technique to improve such information. Recently, the application of this technique to crude hydromethanolic extracts allowed us to demonstrate that individual phenolic biosynthesis differed along the plant growth cycle, reaching its maximal levels in the vegetative stage, and that the extracts were mostly dominated by isoflavones [16]. Najja and coworkers [17] highlighted the DPPH scavenging activity of the crude aqueous and ethanol 70% extracts, but only identified syringic acid and kaempferol through their UHPLC-DAD-ESI-MS^n^ analysis. In another work, when evaluating the antibacterial properties of a *R. raetem* crude extract, Hammouche-Mokrane et al. [18] identified several individual flavonoids and highlighted the dominance of the aglycone forms of isoflavones (genistein and daidzein), along with those of the flavones luteolin and apigenin. It is also of note that only a few purified compounds from *R. raetem* were tested for biological activities. In this respect, the study of Xu et al. [19] and Nur-E-Alam et al. [20] were hallmarks since they isolated for the first time new prenylated, furano and pyrano flavonoids, and simultaneously evaluated their potential to serve as anti-inflammatory agents and/or inhibit alfa-glucosidase activity.

Considering the previous information, studies correlating phytochemical composition and potential biological activities of high-quality extracts (or fractions) of *R. raetem* are necessary to evaluate the exploitation potential of this plant as a source of suitable health promoting agents for use in the food, cosmetic and/or pharmaceutical industries. With this in mind, the main objective of the present study was to monitor the bioactive potential throughout the fractionation of a crude polar extract obtained from *R. raetem* cladodes, in order to expand the existing data reported for this plant in relation to biological properties, as well as variations with the main phytochemicals, caused by purification procedures. As far as we know, this is the first work that evaluates the potency of polar extracts of *R. raetem* towards the activity of amylase (a key enzyme in controlling obesity). Furthermore, considering that the exploitation of *R. raetem*’s cytotoxic effects has been scarcely investigated and limited to the extracts of leaves and flowers, and that antioxidant screening with cell models is currently non-existent, our strategy will further fill these gaps.

## 2. Results and Discussion

### 2.1. Fractionation of R. reatam Methanolic Extract and Phytochemical Composition

**Figure 1:**
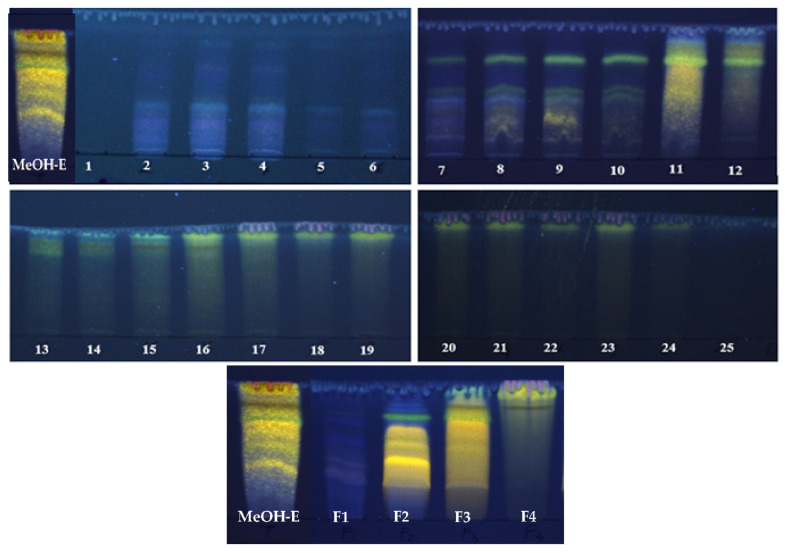
Thin-layer chromatography (revealed by natural products/polyethylenglycol reagent) of the *R. raetam* methanolic extract (MeOH-E), of the 25 fractions obtained by its column fractionation and of the fractions F1–F4 (originated after the combination of distinct chromatographic fractions).

The dried extracts obtained by sequential Soxhlet extraction of *R. raetam* aerial parts with hexane, dichloromethane, methanol and water represented 0.8, 1, 6.5 and 1.42 g/100 g dry plant, respectively. Preliminary studies also indicated that the methanolic extract (MeOH-E) had superior antioxidant abilities than the remaining samples (results not shown), being selected to proceed the work. Its fractionation column yielded 25 fractions with variable chemical profiles, as revealed by TLC analysis (Figure 1). In general, this technique allowed to distinguish different families of compounds (chlorophylls, phenolic aids, flavonoids) when using natural products/polyethylenglycol reagent (NP/PEG) as revealing reagent/UV-lamp. In fact, the 25 chromatograms showed the richness of the obtained fractions in phenolic acids (blue and purple spots) and in flavonoids (green and yellow spots) and, accordingly, we choose to combine samples in four groups: F1 (fractions 1 to 6), F2 (fractions 7 to 10), F3 (fractions 11 to 15) and F4 (fractions 16 to 25). Among those, F3 was the most representative, accounting for approximately 40.90 ± 0.2% of the MeOH-E total mass, followed by F2 > F1 > F4, which corresponded to 24.90 ± 0.12%, 19.80 ± 0.99% and 14.20 ± 0.71%, respectively (Table 1).

Taking into consideration that phenolic compounds are closely associated with several bioactivities (e.g., antioxidant, anti-inflammatory and antitumoral), the *R. raetam* MeOH-E, as well as F1, F2, F3 and F4, were evaluated for their total contents of phenolic compounds (TPC) and of flavonoids (TFC). Overall, data showed that the column fractionation procedure resulted in an enrichment of TPC and TFC in F2 and F3 (342.60 ± 0.04 and 364.80 ± 0.03 of mg gallic acid equivalents (GAE)/g dry residue (DR) and 33.40 ± 0.03 and 35.70 ± 0.18 mg catechin equivalents/g DR, respectively, while F1 and F4 were impoverished in such compounds (Table 1). These results agree with the fact that column fractionation is a suitable technique for purification of phenolic components without loss of sample [21].

**Figure 2:**
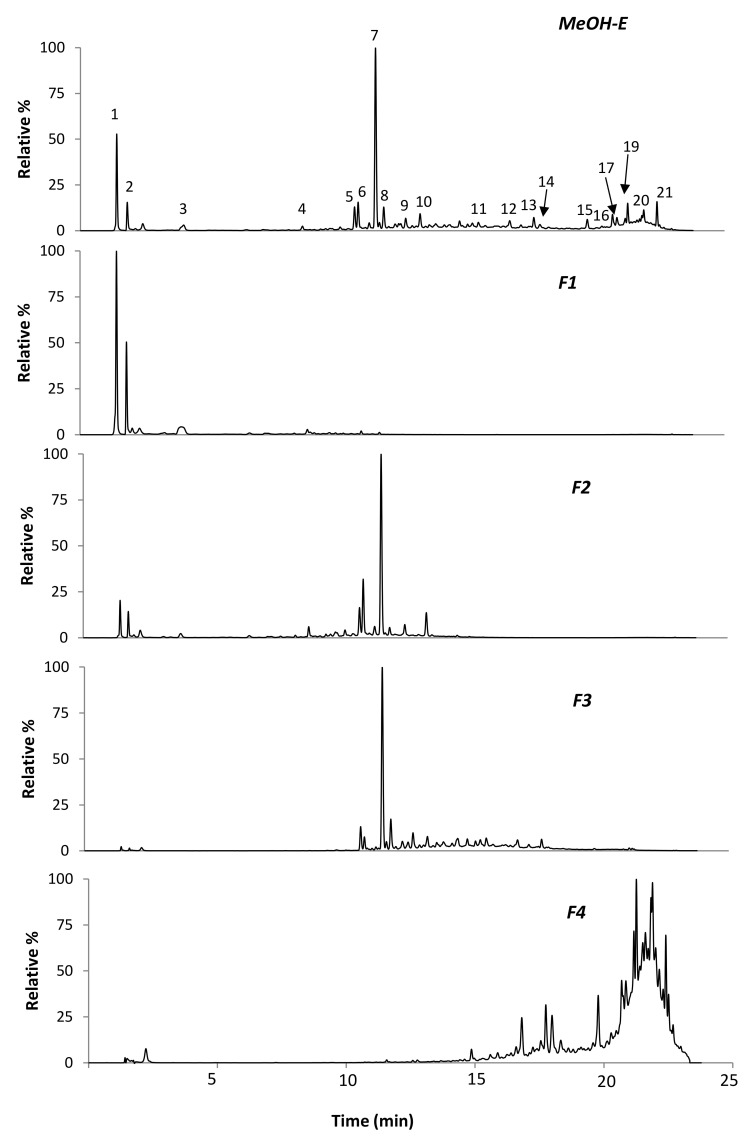
UV chromatographic profiles at 280 nm obtained by LC-DAD-ESI/MS^n^ of *R. raetam* MeOH-E extract and the subsequent fractions F1, F2, F3 and F4. Numbers assigned to the peaks correspond to the peak numbers in Table 2.

In addition, UHPLC-DAD-ESI-MS^n^ analysis was applied for further elucidation of phytochemicals in MeOH-E and F1–F4. As can be concluded from Figure 2 and Table 2, distinct compounds were detected with a relatively well-separation in MeOH-E.

Clearly, this extract was characterized by the predominance of the compound eluted in peak 7 (UV_max_ at 261, 330 nm and a [M-H]^−^ at *m/z* 431), corresponding to genistein-*C*-hexoside, as previously reported [16]. Other MeOH-E prevalent phenolic compounds in this extract were also isoflavones, mostly genistein-*C*-hexoside derivatives, including genistein-*C*-hexoside-*O*-pentoside (peak 6, [M−H]^−^ at *m/z* 431→ 311), genistein-*C*-hexoside-3-hydroxy-3-methylglutaroyl (peak 10, [M−H]^−^ at *m/z* 575→ 431), genistein-*O*-hexoside ie, genistin (peak 9, [M−H]^−^ at *m/z* 431→269) and glycosidic forms of methylated isoflavones, namely calycosin-*O*-hexoside ([M−H]^−^ at *m/z* 445 eluted in peak 5) and tectorigenin-*C*-hexoside ([M−H]^−^ at *m/z* 461 eluted in peak 8). In general, these compounds corresponded to those previously reported by our group in crude MeOH 80% extracts, obtained from *R. raetam* in the vegetative stage [16].

The chromatogram of MeOH-E also revealed minor phenolic compounds that eluted with high percentages of acetonitrile (after 15 min). Besides the isoflavone genistein ([M−H]^−^ at *m/z* 269 eluted in peak 14), the remaining compounds were unusual, mostly likely prenylated or furonaflavonoids. Among them, compounds eluted at RT 15.6, 17.7, 19.8, 20.8, 21.3 and 21.9 (peaks, 11, 13, 15, 17, 19 and 20, respectively) had UV spectra characteristic of flavones with two major absorption peaks (one close to 320 nm and another at 260–270 nm) [22]. Based on phytochemical data reported in literature for *R. raetam*, and on the herein gathered MS^n^ data, the compound eluted in peak 19 ([M−H]^−^ at *m/z* 337→281, 293) was assigned to the prenylated flavone licoflavone C. The main product ions at *m/z* 281 (−56 Da) and at *m/z* 293 (−68 Da) correspond to the loss of C_4_H_8_ and of C_5_H_8_, respectively, which are consistent with the presence of a prenyl chain [23]. Moreover, the compound eluted in peak 15 ([M−H]^−^ at *m/z* 353→283, 282, 335), which showed a fragmentation pattern with a major loss of 70 Da (equivalent to C_4_H_6_O) [24] was assigned to the isoprenylated flavone ephedroidin. Moreover, the compound eluted in peak 17 ([M−H]^−^ at *m/z* 351→333, 293) was assigned to the furanoflavone retamasin B, based on its molecular ion and its fragmentation pattern that showed the main neutral losses of 18 (loss of its OH group) and of 58 Da (loss of C_3_H_6_O) [23]. Note that albeit these compounds have been rarely described in *Retama* spp., they have been previously isolated from *R. raetam* cladodes by Xu et al. [19] on a phytochemical investigation of a CHCl_3_ crude extract. Curiously, regardless not previously being described in *Retama* spp., *O*-hexoside derivatives of ephedroidin and retamasin B in peaks 11 ([M−H]^−^ at *m/z* 515) and 13 ([M−H]^−^ at *m/z* 513), both exhibiting neutral loss of 162 Da in MS^2^ analysis, were also herein detected.

In addition to the above compounds, the MeOH-E also contained other unusual flavonoids which were eluted in peaks 16 ([M−H]^−^ at *m/z* 353) and 13 ([M−H]^−^ at *m/z* 513). The losses of 56 Da (loss of C_4_H_8_) and 70 Da (loss of C_4_H_6_O) registered for the first, suggest that this is a prenylated isomer of the flavone ephedroidin, while the latter most probably represents a *O*-hexoside derivative of the compound eluted in peak 16. Note still that the UV spectra of such compounds do not present a pronounced Band I in their UV spectrum and Band II is close to 280 nm, which is characteristic of a flavane instead of a flavone structure [22]. A more accurate analysis will be requested to complete elucidate such structures in future, as well of other detected ions (e.g., in [M−H]^−^ at *m/z* 365 and [M−H]^−^ at *m/z* 295 eluted in peaks 20 and 21, respectively, and others that were detected in vestigial levels (not shown)). Naturally, the full elucidation of such compounds will require the phytochemical analysis approach to isolate the compounds and their characterization by combined spectroscopic techniques.

Compared to MeOH-E, the chromatographic profile of the purified fractions (F1–F4) was variable. F1 was characterized by the presence of the most polar compounds from MeOH-E, namely organic acids (quinic and citric acids), and the phenolics (piscidic acid eluted in peak 3 and the genistein glycoside eluted in peak 6). On the other hand, the phenolic profile of F2 and F3 revealed close resemblances to each other (even to MeOH-E), although with differences in peak intensities. A main differentiation between these two fractions was observed for flavones which, in opposition to F3, were not detected in F2. In turn, phenolic compounds in F4 were essentially prenylated and furonaflavonoids, corresponding to those eluted in peaks 12–21 of MeOH-E.

### 2.2. Antioxidant Activities

High levels of reactive oxygen species (ROS) are closely associated with the development of most chronic diseases [25]. Natural products, including polyphenols, are known to exert antioxidant properties by means of ROS scavenging, chelating transition metals or modulating the activity of redox-sensitive enzymes [2]. Numerous studies have evaluated the antioxidant activity of *Retama* spp. extracts, most of which thought chemical assays, particularly the 1,1-diphenyl-2-picrylhydrazyl (DPPH) radical scavenging method [2].

The antioxidant ability of MeOH-E and of the four chromatographic resulting fractions (F1, F2, F3 and F4) of *R. raetam* were screened by chemical models, namely through DPPH^•^ and ORAC tests, as well as in a cellular model of fibroblast cells, through monitoring of intracellular ROS levels. The results indicated that the antioxidant potencies of the samples followed the sequence F3> F2 ≃ MeOH-E > F4 > F1, meaning that the purification process allowed the obtaining of more active fractions as compared to the MeOH-E. Indeed, F3 exhibited the lowest inhibitory concentration (IC_50_) value in the DPPH^•^ test, which corresponded to just double of the commercial antioxidant butylated hydroxytoluene (i.e., F3 has half of the BHT activity towards DPPH^•^). In addition, this fraction was the most active against RO_2_^•^ and ROS risings in WS1 cells (Table 3). In general, the values herein found for the antioxidant abilities of F3, F2 and MeOH-E are promising compared to *R. raetam* literature data. As recently revised by Leo’n-Gonza´lez et al. [2], Djeddi and colleagues [11] reported values of IC_50_ for DPPH^•^ between 100 and 1000 µg/mL for aqueous extracts of distinct parts of this plant. Other authors [13] also described lower potencies for hydroalcoholic extracts/purified fractions obtained from cladodes of *Raetam* plants (IC_50_ of 477/33.5 and 252/166 µg/mL for *R. raetam* and *R. sphaerocarpa*, respectively).

Concerning the ability of *Raetam* polar extracts to fight RO_2_^•^, to date, this has been evaluated for aqueous and MeOH-E of *R. sphaerocarpa* fruit [26]. In their study, the authors concluded that among the two extracts, the MeOH-E was the most rich in flavonoids, including the isoflavones daidzein and genistein, and it exerted a noticeable antioxidant activity in oxygen radical-absorbance capacity assay (7.3 µmol trolox/mg), which is close to those obtained by us for MeOH-E and F3 of *R. raetam*.

Regardless, the fractionation process could somewhat increase the ability of ROS scavenging in WS1 cells (F3 vs MeOH-E), the potency of the purified extracts was still much lower than those of commercially available phenolic compounds/antioxidants. The absence of other studies evaluating the potency of *Raetam* crude extracts and/or purified fractions towards ROS precludes any comparison with our results.

### 2.3. Cytotoxicity Assay

Table 4 summarizes the cytotoxicity effects of *R. raetam* MeOH and the subsequent fractions F1–F4 on two tumor cells, specifically from lung (A549) and colon (DLD-1) origins, and the non-tumor human skin fibroblast cells (WS1), according to the resazurin test, which allows estimation of the number of metabolically active cells present in culture. Fractionation of the methanolic extract resulted in a loss of the cytotoxicity activity, with all subsequent fractions exhibiting less activity than that of MeOH-E. This crude extract had promising cytotoxic effects against both the cancerous cells (IC_50_ = 24 ± 2 and 17.5 ± 0.2 μg/mL), being comparatively less toxic to fibroblasts while, among the four fractions, only F3 showed significant efficacy towards the A549 cells, in contrast to the remaining fractions (IC_50_ values were all above 57 ± 5 μg/mL). Hence, possibly, key components and/or synergistic actions relevant for cytotoxic ability were lost during the fractionation of MeOH-E. Even so, it is noteworthy that, in part, isoflavones, and particularly genistein-*C*-hexoside, may act as key players in the cytotoxic effects of F2 and F3. In fact, this isoflavone (major phenolic compound in F2 and F3) was previously reported to inhibit the growth of various cancer cell lines and block carcinogenesis in vitro and in vivo including leukemia, prostate, breast and lung cancer [2,27].

The cytotoxic potential of *R. raetam* crude methanolic extracts from leaves and seeds has been previously tested against large lung carcinoma cell line (COR-L23) [28], for which the authors reported IC_50_ values of 40 and 150 μg/mL, respectively.

### 2.4. Inhibition of Pancreatic α-Amylase Activity

**Figure 3:**
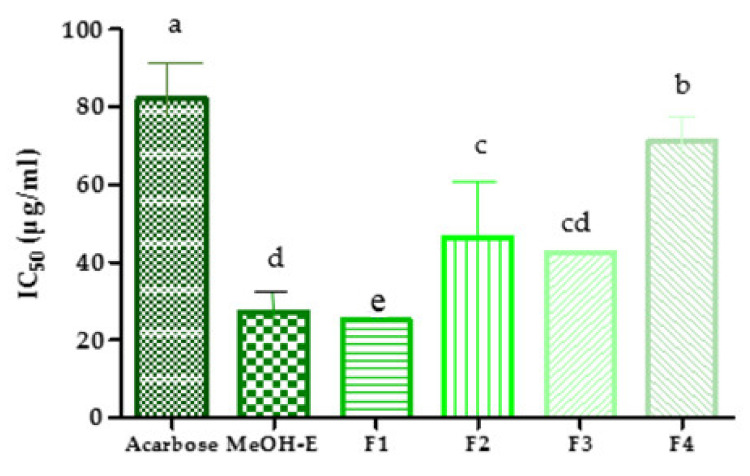
Inhibitory ability of pancreatic α-amylase (IC_50_ in μg/mL) by *R. raetam* methanolic extract (MeOH-E) and the subsequent fractions (F1, F2, F3, F4). Means with different superscripts (a–e) are significantly different at *p* < 0.05.

Pancreatic α-amylase is a key digestive enzyme involved in the metabolism of carbohydrates, which make them important targets for therapeutic control of diabetes and obesity. This enzyme catalyzes the hydrolysis of carbohydrates into simple sugars and since its inhibition hinders the digestion of starch, it contributes to the reduction of postprandial increase in plasma glucose levels [29]. In this study, the ability of *R. raetam* MeOH-E and its subsequent fractions F1–F4 to inhibit the activity of pancreatic α-amylase was assessed *in chemico*. Notably, this capacity was very promising, especially for MeOH-E and F1 (IC_50_ = 27.22 ± 1.33 and 25.12 ± 2.68 µg/mL, respectively), corresponding to approximately 3-fold that of the antidiabetic pharmaceutical drug, acarbose (Figure 3). So, attending to the chromatographic analysis of F1 and MeOH-E, as well as to literature data claiming that quinic acid plays an important role in the inhibition of α-glucosidase and pancreatic α-amylase enzymes [25,29], we may hypothesize that this organic acid may hold a relevant role in the effect of F1 and MeOH-E. Additionally, despite being less effective than MeOH-E and F1, all the remaining fractions (F2, F3 and F4) were still more active than acarbose, showing IC_50_ values of 46.55, 42.52 and 71.05 µg/mL, respectively.

To our knowledge, this is the first study that explores the potential of *Retama* spp. against the activity of α-amylase. Yet, these results are supported by those of Algandaby et al. [26], who demonstrated that the oral administration of a methanolic extract of *R. raetam* fruit to rats caused a reduction in their blood glucose levels. Furthermore, in vitro studies also demonstrated that the extract was capable of inhibiting glucose absorption by rat isolated intestine. No effect was detected in vitro over gluconeogenesis or glycogenolysis or on skeletal muscle glucose uptake [26,30].

### 2.5. Antibacterial Activity

This activity was assessed by measuring the diameter of the growth inhibition zone of five bacterial strains when exposed to MeOH-E or fractions F1–F4. As can be observed in Table 5, the results demonstrated that the antibacterial capacities varied among F1–F4, also depending on the sensitivity of the bacterium. The most relevant antibacterial activities were registered for F4, which effectively inhibited the growth of *Bacillus cereus*, *Staphylococcus aureus* and *Pseudomonas aeruginosa* with an inhibition diameter >12 mm. This ability was superior to that of MeOH-E, especially against Gram+ bacteria. F2 and F3 also displayed some antibacterial activity against *Salmonella typhimurium* (IZ = 10.7 ± 0.5 and 11.7 ± 1.1 mm, respectively) while F1 was inactive against all tested bacteria. As fractionation resulted in a reduction of antibacterial capacity, it is possible that this is due to the loss of relevant compounds and/or of synergetic effects between the various components present in the crude extract.

In fact, the extract of aerial parts of *R. raetam* from Tunisia, rich in flavonoids, tannins and alkaloids, showed significant antibacterial activity against Gram-positive microorganisms, especially methicillin-sensitive and methicillin-resistant Staphylococcus aureus (MSSA, MRSA), thereby suggesting that the use of intermediary polarity solvents is necessary for the extraction of bioactive components with antibacterial activity [27,31].

## 3. Materials and Methods

### 3.1. Plant Material and Fractionation

**Figure 4:**
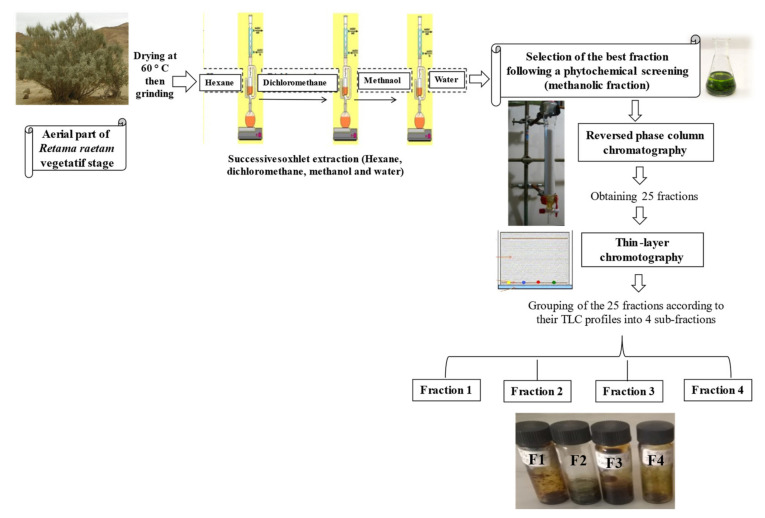
Graphical scheme of the performed processes for the obtaining of *R. raetam* methanolic extract (MeOH-E) and the subsequent fractions (F1, F2, F3, F4).

*R. raetam* samples were collected from the Sebkha of Soliman in December 2015. Plants were identified by the botanist of the Biotechnology Center of Borj-Cedria (CBBC), and a voucher specimen [F-RE 27] was deposited at the Herbarium of the Laboratory of Aromatic and Medicinal Plants. The air-dried and finely grounded shoots (2 kg) were sequentially extracted in a Soxhlet apparatus using several solvents with increasing polarity (hexane, dichloromethane, methanol and water). Each extraction was conducted for 24 h at the boiling point of each solvent. Afterward, the methanolic extract was filtered through a Whatman N°4 filter paper, and solvent was evaporated under reduced pressure using rotary vacuum evaporator. The resulting residue was dissolved in methanol 20% (*v*/*v*) and was fractionated by reversed-phase column chromatography, using the adsorbent resin Diaion HP-20 (Supelco, NJ, USA), and eluted with various methanol-water mixtures of decreasing polarity (methanol 20%, methanol 50%, methanol 80% and methanol 100%) [32]. During fractionation, several fractions were collected from the column (Figure 4). A washing process with pure methanol then with water was carried out to ensure that there were no more compounds left inside the column [32]. The obtained fractions were analyzed by TLC and grouped into four fractions (F1, F2, F3 and F4), based on the similarity of their composition. Samples were reconstituted in appropriate solvents before testing (in pure methanol for DPPH and ORAC tests and UHPLC-DAD-ESI-MS analysis and in DMSO for the remaining tests.

### 3.2. Phenolic Compounds

Contents of total phenolic compounds and flavonoids were determined according to the method of Wasli et al. [33] and the results were expressed as mg of gallic acid or mg catechin per gram of dried residue, respectively. The individual phenolic compounds of the MeOH-E and subsequent fractions were identified by UHPLC-DAD-ESI/MS^n^ following the methodology previously described by Saada et al. [16]. The work was carried out in Ultimate 3000 (Dionex Co., San Jose, CA, USA) apparatus with an ultimate 3000 Diode Array Detector (Dionex Co., San Jose, CA, USA) coupled to a Thermo LTQ XL (Thermo Scientific, San Jose, CA, USA) ion trap mass spectrometer equipped with an ESI source. Analysis was performed on a Hypersil Gold (Thermo Scientific, San Jose, CA, USA) C18 column (100 mm length; 2.1 mm i.d.; 1.9 μm particle diameter, end-capped) and its temperature was maintained at 30 °C. The mobile phase was composed of (A) acetonitrile and (B) 0.1% of formic acid (*v*/*v*). The solvent gradient started with 5–40% of solvent (A) over 14.72 min, from 40–100% over 1.91 min, remaining at 100% for 2.19 min more before returning to the initial conditions. The flow rate was 0.2 mL min^−1^ and UV–V is spectral data for all peaks were accumulated in the range of 200–500 nm while the chromatographic profiles were recorded at 280 and 340 nm.

Control and data acquisition of MS were carried out with the Thermo Xcalibur Qual Browser data system (Thermo Scientific, San Jose, CA, USA). Nitrogen above 99% purity was used, and the gas pressure was 520 kPa (75 psi). The instrument was operated in negative-ion mode with the ESI needle voltage set at 5.00 kV and an ESI capillary temperature of 275 °C. The full scan covered the mass range from *m*/*z* 100 to 2000. CID–MS/MS and MS^n^ experiments were simultaneously performed for precursor ions using helium as the collision gas with a collision energy of 25–35 arbitrary units [34]. The quantification of isoflavones in the extracts was obtained using ginestin (y = 11699.79𝑥 + 33,085.50; R2 = 0.999), being the results for each target phenolic compound expressed in equivalents of ginestin.

### 3.3. Antioxidant Activities

#### 3.3.1. DPPH Quenching Ability

The radical scavenging activity of the MeOH-E and subsequent fractions towards DPPH followed the general procedure of Wasli et al. [35]. For this, 1 mL of the extracts at various concentrations was mixed with 0.25 mL of a DPPH-methanolic solution (0.2 mM) and allowed to react in the dark for 30 min. The absorbance of the resulting solution was read at 517 nm. The antiradical activity was determined using this formula:

Inhibition (%) = [(A0 − A1)/A0] × 100

where A0 is the absorbance of the control at 30 min and A1 is the absorbance of the sample at 30 min. The antiradical activity was expressed as IC_50_ (µg/mL).

#### 3.3.2. ORAC Assay

The procedure was adapted from the method described by Wasli et al. [36]. The ORAC assay was accomplished in black round bottom 96-well microplates (Costar) on a Fluoroskan Ascent FL™ plate reader (Labsystems) using an automated injector. The test was directed at 37.5 °C and in pH 7.4 phosphate buffer, with a blank sample in parallel. Trolox was used as standard control at different concentrations and in quadruplicate. A gradient of 16 concentrations of the extracts was set without replication. The fluorimeter was planned to register the fluorescence (λ ex.: 485 nm/em.: 530 nm) each minute, once adding 375 mM of 2,2-azobis (2-amidinopropane) dihydrochloride (AAPH), for 35 min. The final results were calculated using the net area under the curves of the extract concentrations for which reduction of at least 95% of fluorescence was detected at 35 min and which also showed a linear dose–response pattern. ORAC values were stated in micromoles of Trolox equivalents (TE) per gram (μmol TE/g).

#### 3.3.3. Antioxidant Cell Assay Using 2′,7′-Dichlorofluorescin-Diacetate (DCFH-DA)

Antioxidant activity was assessed via the DCFH-DA test as reported by Legault et al. [37], with modifications. Human skin fibroblast cells were plated in 96 microwell plates at 10,000 cells per well and incubated for 24 h at 37 °C and 5% CO_2_. Next, the cells were washed with 150 μL Hank’s balanced salt solution (HBSS) at pH 7.4 and incubated for 30 min with 100 μL HBSS (pH 7.4) holding 5 μM DCFH-DA (Sigma–Aldrich). Then, the cells were washed with 150 μL HBSS.. To assess antioxidant activity, the cells were incubated either with a growing concentration of samples from *R. raetam*, quercetin or Trolox, in the presence or absence of 200 mΜ tert-butylhydroperoxide (t-BuOOH). The final concentration of solvent in the culture medium was preserved at 0.5% (*v*/*v*) to evade solvent toxicity Fluorescence was quantified immediately after t-BuOOH addition (T0) and after 4 h of incubation, with an automated 96-well plate reader (Fluoroskan Ascent FL™, Labsystems) via an excitation wavelength of 485 nm and an emission wavelength of 530 nm. The oxidation dose-response was obtained with the fluorescence increase from T0 to 4 h for each tested condition.

### 3.4. Cytotoxicity Assay

The human lung carcinoma A549 (ATCC #CCL-185) and colon adenocarcinoma DLD-1 (ATCC #CCL-221) cell lines were purchased from the American Type Culture Collection (ATCC, Manassas, VA, USA). The A549 and DLD-1 cell lines were developed in Minimum Essential Medium with Earle’s salts. The media was complemented with 10% fetal calf serum (Hyclone, Logan, USA) for (A549 and DLD-1), solution of vitamins (1×), sodium pyruvate (1×), nonessential amino acids (1×), penicillin (100 IU) and streptomycin (100 μg/mL) (Mediatech Cellgro). Cells were cultivated in a humidified atmosphere at 37 °C in 5% CO_2_.

Exponentially growing cells were plated at a density of 5 × 10^3^ cells per well in 96-well microplates (Costar, Corning Inc., Corning, NY, USA) in 100 μL of culture medium and were permitted to paste for 16 h before treatment. Then, 100 μL of growing concentrations of sample dissolved in the suitable solvent (DMSO) were added. The cells were incubated for 48 h in the presence or in the absence of sample. Cytotoxicity was measured by means of the resazurin reduction test as reported by O’Brien et al. [38]. The final concentration of solvent in the culture medium was preserved at 0.5% (*v*/*v*) to evade solvent toxicity. Fluorescence was quantified on an automated Fluoroskan Ascent Fl™ plate reader (Labsystems) with an excitation wavelength of 530 nm and an emission wavelength of 590 nm. Cytotoxicity was stated as the concentration of extract inhibiting cell growth by 50% (IC_50_).

### 3.5. Pancreatic α-Amylase Activity In Vitro Study

The α-amylase inhibitory activity was performed by the method described by Pereira et al. [39], with slight modification. Two hundred µL of varying concentration of MeOH-E and subsequent fractions dissolved in 20 mM, pH 6.9 phosphate buffer was added to 400 µL of a 0.8% (*w*/*v*) starch solution in the same phosphate buffer. Experimental tubes were incubated at 37 °C for 10 min, and the reaction was started with the addition of 200 µL of α-amylase solution which then kept at 37 °C for 5 min. After that, the reaction mixture was collected and instantaneously mixed with 600 µL dinitrosalicylic acid reagent (1.0 g of 3.5-dinitrosalicylic acid in 20 mL of 2 M NaOH + 50 mL distilled water + 30 g potassium sodium tartrate tetrahydrate). A total of 200 µL of the second aliquot was further collected 15 min later and mixed with dinitrosalicylic acid reagent as well. Samples were then boiled in a water bath (100 °C) for 10 min and, once they had cooled, 250 µL were transferred to each well in a 96-well microplate. Absorbance was measured at 540 nm a using an ELX800 microplate reader. The concentration of acarbose and of plant MeOH-E and subsequent fractions required to inhibit 50% of α-amylase activity under the conditions was defined as the IC_50_ value.

### 3.6. Antibacterial Activity

The antibacterial activity was assessed by the agar disk diffusion method [40] against five human pathogenic bacteria: Gram-positive bacteria *Bacillus cereus* ATCC 14579 and *Staphylococcus aureus* ATCC 25923 and Gram-negative bacteria including *Salmonella typhimurium* ATCC 1408, *Pseudomonas aeruginosa* ATCC 27853 and *Escherichia coli* ATCC 85218. The bacterial strains were first grown on Muller Hinton medium at 37 °C for 24 h prior to seeding onto the nutrient agar. One or several colonies of the indicator bacteria were transferred into API suspension medium (BioMérieux, Marcy-l’Étoile, France) and adjusted to the 0.5 McFarland turbidity standard with a Densimat (BioMérieux, Marcy-l’Étoile, France). A sterile filter disc with 6 mm diameter (Whatman paper N°3) was placed on the infusion agar seeded with bacteria, and 10 µL per disc of the extract and standards Gentamicin (100 µg/mL) were added. The treated Petri dishes were incubated at 37 °C for 24 h. The antibacterial activity was determined by measuring the zone of growth inhibition surrounding the discs.

### 3.7. Statistical Analysis

Data were analyzed using one-way ANOVA followed by Tukey’s post-hoc test. The statistical tests were applied using GraphPad Prism, version 6, and the significance level was *p* < 0.05.

## 4. Conclusions

The fractionation of *R. raetam* methanolic extract on open-column chromatography yielded four fractions (F1, F2, F3 and F4). The obtained results allowed us to distinguish F3 as the promising fraction which exhibited noteworthy in vitro biological activities particularly against peroxyl radicals. In turn, F1 revealed a potent capacity to inhibit pancreatic α-amylase activity. Overall, these data proved the strong potentialities of *R. raetam* fractions to serve as a source of phytochemical compounds, particularly derivatives of the isoflavone genistein and of organic compounds that can be applied as bioactive agents. In the future, it will be essential to isolate the main phenolic compounds in each fraction to fully elucidate their structure and directly correlate them with the bioactivities studied here. In addition, it will also be very interesting to understand whether there are synergistic and/or antagonistic effects on MEOH-E and on each fraction.

## Figures and Tables

**Table 1:** Total contents of phenolic compounds (TPC) and flavonoids (TFC) of *Retama raetam* methanolic extract (MeOH-E) and the subsequent fractions (F1, F2, F3, F4).

Samples	Yield (%)	TPC (mg GAE/g DR)	TFC (mg Catechin/g DR)
MeOH-E	-	148.20 ± 0.74 ^b^	11.50 ± 2.14 ^b^
F1	19.80 ± 0.99 ^c^	52.00 ± 0.05 ^c^	2.12 ± 0.01 ^d^
F2	24.90 ± 0.12 ^b^	342.60 ± 0.04 ^a^	33.40 ± 0.03 ^a^
F3	40.90 ± 0.2 ^a^	364.80 ± 0.03 ^a^	35.70 ± 0.18 ^a^
F4	14.20 ± 0.71 ^d^	48.27 ± 0.06 ^d^	4.20 ± 0.12 ^c^

Values of TPC and TFC are expressed as mg gallic acid equivalents (GAE) or mg catechin equivalents per g of dried residue (DR), respectively. Means in the same column followed by different letters are significantly different at *p* < 0.05.

**Table 2:** UHPLC-DAD-ESI-MS^n^ analysis of *R. raetam* MeOH-E extract and subsequent fractions F1, F2, F3 and F4 obtained after column chromatography purification.

Peak	RT (min)	λmax (nm)	*m*/*z*	ESI-MS^n^	Proposed Compound	MeOH-E	F1	F2	F3	F4
1	1.4	226, 304	191	MS^2^ [191]: 127, 173, 111, 85, 93	Quinic acid	+	+	+	-	-
2	1.8	224, 302	191	MS^2^−[191]: 111, 173	Citric acid	+	+	+	-	-
3	3.9	275	255	MS^2^ [255]: 165, 193	Piscidic acid	+	+	+	-	-
4	8.8	260, 330sh	593	MS^2^ [593]: 473, 311	Genistein-*C*-hexoside-*O*-hexoside	30.6 ± 1.45 ^b^	-	32.4 ± 3.65 ^a^	23.2 ± 1.44 ^c^	-
5	10.6	255, 315sh	445	MS^2^ [445]: 283; MS3 [283]: 268	Calycosin-*O*-hexoside	32.2 ± 2.34 ^a^	-	11.4 ± 0.67 ^c^	29.4 ± 0.13 ^b^	-
6	10.9	261, 330sh	563	MS^2^ [563]: 311, 283, 341, 431	Genistein-*C*-hexoside-*O*-pentoside	17.39 ± 2.33 ^c^	0.76 ± 0.34 ^d^	64.08 ± 0.12 ^a^	33.00 ± 0.16 ^b^	-
7	11.6	261, 330sh	431	MS^2^ [431]: 311	Genistein-*C*-hexoside	157.3 ± 1.15 ^c^	-	207.6 ± 2.21 ^b^	241.6 ± 0.76 ^a^	-
8	11.8	262, 330sh	461	MS^2^ [461]: 341, 371	Tectorigenin- *C*-hexoside	+	-	+	+	-
9	12.7	260, 321sh	431	MS^2^ [431]: 296, 268	Genistin	8.79 ± 0.77 ^c^	-	23.2 ± 0.47 ^a^	23.2 ± 0.21 ^a^	-
10	13.2	262, 328sh	575	MS^2^ [575]: 431, 311, 341	Genistein-*C*-hexoside-3-hydroxy-3-methylglutaroyl	7.19 ± 1.09 ^c^	-	28.2 ± 0.22 ^a^	18.6 ± 0.17 ^b^	-
11	15.6	271, 325	515	MS^2^ [515]: 353, 395	Ephedroidin-*O*-hexoside	+	-	-	+	+
12	16.8	282	513	MS^2^ [513]: 351, 485	ND	+	-	-	+	+
13	17.7	269, 300, 325	513	MS^2^ [559]: 351	Retamasin β-*O*-hexoside	+	-	-	+	+
14	18	261, 320sh	269	MS^2^ [269]: 269	Ginestein	+	-	-	+	+
15	19.8	272, 326	353	MS^2^ [353]: 283, 282, 335	Ephedroidin	+	-	-	-	+
16	20.7	281	353	MS^2^ [353]: 283, 297	ND	+	-	-	-	+
17	20.8	259, 300, 323	351	MS^2^ [351]: 333, 293	Retamasin B	+	-	-	-	+
18	21.2	277	497	MS^2^ [497]: 335	ND	+	-	-	-	+
19	21.3	271, 320	337	MS^2^ [337]: 281, 293	Licoflavone C	tc	-	-	-	+
20	21.9	272, 321	365	ND	ND	+	-	-	-	+
21	22.4	275	295	ND	ND	+	-	-	-	+

Levels of phenolic compounds are in mg/g of dried residue; ND: not determined; tc: traces − absent; +: present; sh: shoulder in the UV spectrum. With exception of genistin that was confirmed with a commercial standard, the remaining compounds were identified based on literature data and/or interpretation of their fragmentation profile [16,22,23,24], as described in the body of the manuscript. Means in the same line with different superscripts are significantly different at *p* < 0.05.

**Table 3:** Ability of *R. raetam* MeOH-E and the subsequent fractions F1, F2, F3 and F4 to scavenge DPPH^•^ and RO_2_^•^, and to reduce intracellular levels of reactive oxygen species in WS1 cells.

Samples	DPPH^•^ (IC_50_ µg/mL)	ORAC (µmol trolox/mg)	ROS in WS1 Cells (IC_50_ µg/mL)
MeOH-E	37 ± 1.85 ^c^	6.61 ± 0.42 ^d^	6.0 ± 2.0 ^c^
F1	280 ± 1.4 ^a^	1.13 ± 0.25 ^f^	20.0 ± 2.0 ^a^
F2	35 ± 1.75 ^c^	4.74 ± 0.51 ^e^	6.2 ± 0.6 ^c^
F3	22 ± 1.1 ^d^	8.10 ± 0.25 ^c^	4.5 ± 0.7 ^d^
F4	105 ± 0.52 ^b^	1.36 ± 0.25 ^f^	10.0 ± 0.8 ^b^
Trolox	-	-	0.021 ± 0.002 ^f^
Quercetin	-	21.41 ± 3.49 ^b^	0.090 ± 0.004 ^f^
Catechin		26.87 ± 1.99 ^a^	0.20 ± 0.03 ^e^
BHT	11.50 ± 0.57 ^e^	-	-

BHT; butylated hydroxytoluene. Means in the same column with different superscripts are significantly different at *p* < 0.05.

**Table 4:** Cytotoxic activity of *R. raetam* methanolic extract (MeOH-E) and the subsequent fractions (F1, F2, F3, F4) against tumor (A549, DLD-1) and healthy (WS 1) cell lines.

	IC_50_ (μg/mL)		
Samples	A549	DLD-1	WS1
MeOH-E	24 ± 2 ^e^	17.5 ± 0.2 ^d^	153 ± 30 ^b^
F 1	92 ± 13 ^b^	>200 ^a^	143 ± 16 ^c^
F 2	>200 ^a^	>200 ^a^	>200 ^a^
F 3	34 ± 2 ^d^	104 ± 8 ^c^	106 ± 8 ^e^
F 4	57 ± 5 ^c^	123 ± 8 ^b^	115 ± 10 ^d^
Etoposide	18.2 ± 4.0 ^f^	4.6 ± 0.3 ^e^	15.3 ± 7.0 ^f^

Etoposide was used as positive control. Means in the same column with different superscripts are significantly different at *p* < 0.05.

**Table 5:** Antibacterial activity of *R. raetam* MeOH-E, fractions F1, F2, F3, F4 and antibiotic (at 100 µg/mL), against five human pathogenic bacteria. Inhibition zone (IZ) calculated as diameter around the disc (mm).

		Diameter of the Inhibition Zone Including That of the Disk (mm ± SD)					
Bacterial Strains	Source No.	F1	F2	F3	F4	MeOH-E	Gentamicin
Gram+							
*Bacillus cereus*	ATCC 14579	-	-	-	15.0 ± 0.6 ^b^	12.0 ± 0.6 ^c^	28.0 ± 1.4 ^a^
*Staphylococcus aureus*	ATCC 25923	-	-	9.0 ± 0.0 ^d^	17.0 ± 0.5 ^b^	15 ± 1.0 ^c^	32.7 ± 1.6 ^a^
Gram-							
*Escherichia coli*	ATCC 25922	-	-	-	-	17.00 ± 0.50 ^a^	15.00 ± 0.75 ^b^
*Pseudomonas aeruginosa*	ATCC 27853	-	-	-	12.0 ± 0.0 ^c^	16.0 ± 0.0 ^b^	30.33 ± 1.51 ^a^
*Salmonella typhimurium*	ATCC 1408	-	10.7 ± 0.5 ^d^	11.7 ± 1.1 ^c^	-	14.0 ± 0.5 ^b^	21.00 ± 1.05 ^a^

IZ: inhibition zone: SD: standard deviation. The diameter of disc was 6 mm. No antimicrobial activity (–), inhibition zone <7 mm. Weak inhibition zone, inhibition zone 7 mm. Slight antimicrobial activity, inhibition zone 8 to 9 mm. Moderate antibacterial activity, inhibition zone 10 to 11 mm. High antibacterial activity, inhibition zone 12 to 14 mm. Strong antibacterial activity, inhibition zone >14 mm. In each line, means followed by the same letter are not significantly different at *p* < 0.05.

## Data Availability

Data is contained within the article.

## Sample Availability

Samples of the compounds are not available from the authors.
